# Aerogels as Carriers for Oral Administration of Drugs: An Approach towards Colonic Delivery

**DOI:** 10.3390/pharmaceutics15112639

**Published:** 2023-11-17

**Authors:** Carlos Illanes-Bordomás, Mariana Landin, Carlos A. García-González

**Affiliations:** AerogelsLab, I+D Farma Group (GI-1645), Department of Pharmacology, Pharmacy and Pharmaceutical Technology, iMATUS and Health Research Institute of Santiago de Compostela (IDIS), Universidade de Santiago de Compostela, E-15782 Santiago de Compostela, Spain; carlosjavier.illanes@rai.usc.es

**Keywords:** aerogels, porous systems, colonic drug delivery, polysaccharides, oral administration, inflammatory bowel diseases

## Abstract

Polysaccharide aerogels have emerged as a highly promising technology in the field of oral drug delivery. These nanoporous, ultralight materials, derived from natural polysaccharides such as cellulose, starch, or chitin, have significant potential in colonic drug delivery due to their unique properties. The particular degradability of polysaccharide-based materials by the colonic microbiota makes them attractive to produce systems to load, protect, and release drugs in a controlled manner, with the capability to precisely target the colon. This would allow the local treatment of gastrointestinal pathologies such as colon cancer or inflammatory bowel diseases. Despite their great potential, these applications of polysaccharide aerogels have not been widely explored. This review aims to consolidate the available knowledge on the use of polysaccharides for oral drug delivery and their performance, the production methods for polysaccharide-based aerogels, the drug loading possibilities, and the capacity of these nanostructured systems to target colonic regions.

## 1. Introduction

The oral administration route is the most common approach for the local and systemic therapeutic treatments of a wide range of pathologies [1,2,3]. It is the natural physiological pathway to incorporate nutrients into the body and the easiest way to administer drugs. Active compounds can be absorbed in three different sections of the gastrointestinal tract (GIT): the stomach, small intestine, and large intestine. The stomach is a structure specialized in decompounding the ingested food, but its capability for the absorption of drugs is limited. The small intestine is the section specialized in nutrient absorption due to its tremendous surface, where the drugs can easily penetrate by paracellular transport to the systemic circulation. Finally, in recent decades, the colon has been postulated as an area of high interest for drug delivery. The physiological characteristics of the colon and the high residence times in this area facilitate absorption, especially for drugs that are degradable by intestinal enzymes. Furthermore, colonic administration allows the local treatment of certain pathologies such as inflammatory bowel diseases (IBD) or colorectal cancer [4,5,6].

Successful colonic administration requires protection of the drug against different pHs, enzymes, microorganisms, or peristaltic movements through the GIT [6,7]. Currently, capsules or tablets with pH-dependent, pressure-dependent, or time-modified release coatings are used for colonic delivery. Once in the colon, the drug should be released from the delivery system at specific rates and solubilized in the tissues, avoiding toxic effects and getting an optimum pharmacological response [6,8]. Unfortunately, there are great intra-individual and inter-individual variabilities due to the gastric and intestinal transit times, volumes of liquid through the GIT, diet, food–drug interactions, pathologies, gender, or age of patients that compromise the efficacy of the formulations [9].

Polysaccharides have been proposed as the main excipients for colonic formulations, as coating materials, matrices, hydrogel precursors, or prodrug ingredients [6,10,11]. Their physicochemical properties confer on them enzymatic and/or pH resistance. This allows polysaccharides to pass unaltered through the GIT, thus protecting the drug. Then, once in the colonic area, polysaccharides are decomposed by a microbiota composed of a huge number of microorganisms (10^11^ CFU/g fecal content), allowing the complete release of the drug [10,11]. Polysaccharides also have some industrial advantages, like their abundance and low price, biodegradability, and non-toxicity for the environment [11,12,13].

Hydrogels can be formed from polysaccharides by physical or chemical crosslinking, giving rise to three-dimensional systems of high porosity that can host and protect drugs and then release them in response to different stimuli [14,15,16]. Dry gels are advantageous over hydrogels in terms of drug stability as they are less susceptible to microbiological and chemical degradation [17]. Polysaccharide-based hydrogels can be dried using various methods, and their choice extraordinarily affects the structural properties of the resulting dry gel. Heat drying results in xerogels with significant structural shrinkage, and freeze-drying yields cryogels suitable for certain pharmaceutical applications like oral disintegrating formulations (e.g., ODTs) or scaffolds [18,19]. Supercritical fluid (SCF) drying produces dry gels with a preserved porous nanostructure (the so-called aerogels) and different formats and sizes depending on the chosen preparation technique [20]. 

Aerogels are dry solids with extremely low weight and bulk density (<0.2 g/m^3^), high porosity (>90%) predominantly in the mesoporous range (2–50 nm), and high specific surface area (>200 m^2^/g) [20,21]. They can be prepared from a wide range of inorganic and organic sources, including polysaccharides. The initial mesoporosity of the wet structures is preserved in the aerogels, which gives them a high surface area and excellent drug-loading capacity in the amorphous state [20]. A significant proportion of newly discovered New Molecular Entities (NME) fail to realize their full clinical potential, primarily because of their limited aqueous solubility, stability issues, and, in many instances, inadequate tissue targeting properties. The possibility of loading and stabilizing drugs in aerogels in an amorphous state opens new perspectives for the development of pharmaceutical formulations with poorly soluble NME. Its incorporation into mesoporous structures that remain in a metastable state would improve its dissolution rate, overcoming this problem [22].

A number of aerogel formulations for drug delivery have been recently investigated and proposed for different applications, such as pulmonary drug delivery [23], wound healing [24], or oral administration of drugs [20]. Various polysaccharides, such as alginate, pectin, or starch, among others, processed using various technologies such as emulsification or drip-gelation, have demonstrated their usefulness in producing aerogel particles that can incorporate drugs in their amorphous state. Furthermore, they exhibit modified drug release profiles, offering promising opportunities for enhancing drug absorption and stability [22,25,26].

The enzymatic- and/or pH-resistance of certain polysaccharide aerogels also makes them potential candidates for the development of colonic delivery dosage forms [6]. This approach is attractive for delivering biologics like peptides, given their reduced susceptibility to proteolytic degradation in the colon compared to other sections of the GIT. Additionally, the extended residence times and favorable pH levels in this region contribute to improved bioavailability [27,28]. However, the requirements of colonic formulations are higher than those of conventional oral dosage forms (burst release) [15]. The release of the drug must be specific and controlled in the colonic area. Therefore, the formulation must bypass the aforementioned adverse conditions of the GIT until reaching the colon and then release the drug at the appropriate rate to ensure that no toxic effects or problems of therapeutic inefficacy occur [29].

This review explores recent advances in porous polysaccharide-based formulations for oral drug delivery, providing a critical assessment of aerogels’ potential for colonic drug delivery for the first time. The sections of this article will cover an overview of the GIT characteristics with a special focus on the large intestine, the selection of suitable polysaccharides (alginate, chitosan, pectin, cellulose, starch, and glucomannan, among others) for colonic drug delivery, as well as the analysis of formulation strategies for aerogel design and prior art on aerogels with potential colonic application, including their preparation methods. Finally, alternative technological approaches for producing aerogels with appropriate properties for colonic drug delivery will be presented as future perspectives.

## 2. Gastrointestinal Conditions: An Overview and Essential Considerations

**Figure 1:**
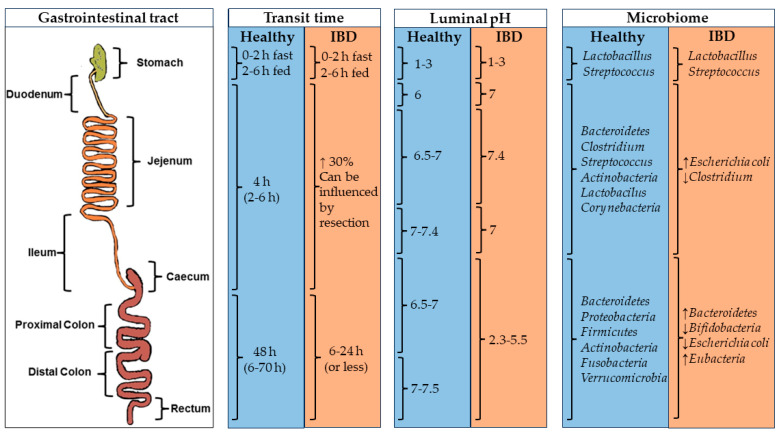
Physiological factors (transit time, luminal pH, and microbiome) in the GIT of healthy and IBD patients influence oral drug delivery. Image adapted from [3].

Digestion through the GIT is a complex process involving enzymes, pH ranges, microorganisms, and movements designed to process food and absorb nutrients. The pH values, microbiomes, and transit times are shown in Figure 1, and the main enzymes implicated in the digestion are disclosed in Table 1. Oral dosage forms for colonic drug delivery need to withstand adverse conditions throughout the GIT until reaching the colon and then releasing the drug payload into the tissues before being eliminated [3].

Each section within the GIT is characterized by a different pH, residence time, ionic force, enzymatic conditions, and gut pressure [3,11]. The upper GIT starts in the mouth, where the food components are chewed and mixed. After that, the food is transported to the stomach through the pharynx and the esophagus. In the stomach, different mixing movements and the low pH (approximately 1–3) allow the degradation of the food content. The residence time of the compounds in the stomach depends on the diet and the gastric state (fast state: 0–2 h; fed state: 2–6 h). After that, the chyme is transported from the stomach to the duodenum, where it is digested by many enzymes at a pH of ca. 6 [3,30,31].

The lower GIT comprises the rest of the small intestine (i.e., jejunum and ileum) and the large intestine [3]. In the small intestine, the efficient absorption of digested nutrients is facilitated by its substantial surface area, which, in healthy individuals, allows for a residence time of 4 h. The pH values in this region are around 7. Finally, in the colon, non-digestible food undergoes water and nutrient processing, with fecal content moving slowly through the colon segments. Bacterial activity in the large intestine, driven by enzymes not produced by the human body, degrades polysaccharides, fatty acids, and active compounds. This process usually takes around 48 h but can last up to 70 h in some cases and is shortened to 6 h or less in pathological conditions. Finally, residues accumulate in the rectum and are expelled as stools [30,32]. pH levels in the cecum and ascending colon range from approximately 6.5 to 7.0. As transit progresses through the large intestine, pH levels increase to 7.5 in the sigmoid colon and rectum. Generally, the pH values tend to be lower in pathological states compared to healthy conditions.

**Table 1:** Digestive enzymes are present in the human GIT and classified by substrate type.

Type of Nutrient (Substrate)	Enzyme	Section of GIT	References
Polysaccharides and oligosaccharides	α-amylase (lingual and pancreatic)	Oral cavity and duodenum	[3]
	Oligosaccharidases	Small intestine	[3]
	β-D-galactosidases	Colon	[33]
	β-D-xilosidases	Colon	[33]
	α-L-arabinofuranosidase	Colon	[33]
Lipids	Lipase (lingual, gastric and pancreatic)	Oral cavity, stomach, duodenum	[3,34]
	Colipase	Duodenum	[3,34,35]
Proteins and aminoacids	Pepsin (pepsinogen)	Stomach	[35]
	Enteropeptidases	Small intestine	[3,34]
	Trypsin (trypsinogen)	Duodenum	[34,35]
	Chymotrypsin	Duodenum	[34,35]
	Elastase	Duodenum	[34,35]
	Carboxypeptidases (A and B)	Duodenum	[34,35]
Other	Azoreductases	Colon	[33]
	Nitroreductases	Colon	[33]

Recently, the microbiota has gained special attention due to its impact on the therapeutic activity of certain treatments [36,37,38] and its potential involvement in the development of chronic diseases, such as IBD [39], Parkinson’s [40], or Alzheimer’s [41]. GIT sections are colonized by different types of microorganisms. From a digestive perspective, the microbiota plays a crucial role in breaking down components that resist digestion in preceding sections of the GIT due to enzymatic systems. Also, certain microbiota metabolites, such as short-chain fatty acids, serve as a source of energy for enterocytes [3,42].

Dietary choice influences the microbiota [42]. While microorganisms inhabit the entire GIT from mouth to anus, the major microbial population grows under anaerobic conditions in the colon. The mouth microbiota has a remarkable diversity of microorganisms (>1000 species), including *Bacteroidetes*, *Proteobacteria*, *Streptococcus*, *Escherichia*, *Clostridium*, *Firmicutes*, *Actinobacteria*, *Spirochaetes*, and *Fusobacteria*, the main phyla. The microbiota variety and population decrease in the stomach due to the enzymatic activity and the low pH. However, the bacterial population gradually increases again until it reaches the colon [43]. 

In the colon, the bacterial population increases significantly, reaching levels of up to 1 × 10^11^ CFU/g of wet content. Assuming a colon volume above 400 mL and a wet content density of 1 g/mL, the number of bacteria in the total colon volume would be around 4 × 10^14^ units [44]. The main species in this area are *Bacteroidetes*, *Proteobacteria*, *Firmicutes*, *Actinobacteria*, *Fusobacteria*, and *Verrucomicrobia* spp. [3,31,42]. Although the bacterial population remains relatively constant throughout the colon within individual patients, there is substantial interpersonal variability in terms of biodiversity. Nevertheless, microbial functions remain remarkably consistent among different patients [42].

Colonic bacteria, in symbiosis with their host, produce numerous enzymes that decompose dietary polysaccharides and peptides into lactate and short-chain fatty acids (SCFAs). Colonocytes use these SCFAs for energy and metabolism [45,46,47]. Key enzyme groups include glycoside hydrolases and the polysaccharide lyases primarily produced by the *Bacteroidetes* and *Firmicutes* bacteria phylum [45,46,48]. The Azoreductase family, which specializes in clearing azo groups, is also significant and has potential applications in prodrug development, ensuring drug release specifically in the colonic area [49].

GIT fluids should be particularly taken into account when designing colonic forms. These fluids vary in volume and composition based on multiple factors such as GIT section, diet, or GIT state (fed or fasted) [47,50]. Generally, stomach fluid content is higher in the fed state (686 mL) and decreases during digestion (45 mL). In the small intestine, the fasting state has more water (105 mL) compared to the postprandial state (54 mL). Finally, in the colon, the fasting state has slightly more liquids (13 mL) than the fed state (11 mL) [50,51]. Factors like the number of pockets of liquid in the colon, their water content, and their distribution significantly affect drug administration in the colonic region, as they influence the disintegration and dissolution processes of the dosage forms [50,51].

## 3. Polysaccharides as Carriers for Colonic Delivery

Several strategies have been explored to achieve colonic drug delivery with oral dosage forms [6,7,32]. An effective approach involves the use of polymers capable of withstanding pH variations across the GIT (pH 1 to 7) to ensure the release of the drug, specifically in the colon. These polymers include a wide range of derivates of acrylic and methacrylic acids (markets like Eudragits^®^), or derivates of cellulose, like hydroxypropyl methylcellulose phthalate (HPMCP), soluble with pHs above 5.5, or cellulose acetate phthalate, also called cellacefate, soluble with pHs above 6.2 [52,53,54]. Another option is the use of time-release dosage forms, given the extended colon residence time compared to other segments of GIT [7,55]. Extensive research has also been conducted on the use of prodrugs to reach the colon, avoiding absorption in the upper GIT. Prodrugs are drugs covalently bound to a ligand involving various hydrophilic and lipophilic functional groups or specific sites on cellular transporters. After their administration, the active compound is activated through the cleavage of the bond between the ligand and the drug mediated by an enzymatic process that involves hydrolytic enzymes (such as carboxylesterases, azoreductases, or phosphatases), oxidoreductases, transferases, and liases from various colonic bacteria [56,57]. Another suitable technological alternative is the use of resistant polymers against human enzymes to retain the drug content until reaching the colon, where a diverse range of bacterial enzymes can digest the dosage forms [7].

Some polysaccharides are excellent candidates as polymeric excipients for specific colon treatments due to their resistance to gastrointestinal enzymes. The unique glycoside bonds of polysaccharides prevent degradation by gastrointestinal enzymes, allowing the polysaccharide-based dosage forms to reach the large intestine intact. There, the enzymatic system of the colon microbiota breaks the glycoside bonds, facilitating drug release. This property gives them suitable characteristics to produce various forms of colonic administration, including coated tablets, multiparticulate systems, and hydrophilic matrix tablets [4,11,47].

Polysaccharides are readily available from natural resources such as plants, algae, animals, or microorganisms (Table 2), and they can be easily modified to suit specific needs [58,59]. These biodegradable biopolymers are recognized as GRAS (Generally Recognized As Safe) materials by the FDA (Food and Drug Administration) and offer versatility in formulating drug delivery systems for the colon.

### 3.1. Alginate (Alg)

Alginate is a linear anionic polysaccharide (Table 2) known for its ability to form gels when crosslinked with different divalent and trivalent cations (affinity order is Pb^2+^ > Cu^2+^ > Cd^2+^ > Ba^2+^ > Sr^2+^ > Ca^2+^ > Fe^3+^ > Co^2+^, Ni^2+^, and Mn^2+^). Among them, calcium cation is the most used due to its good affinity and low toxicity [17,73]. The crosslinking process relies on coordination bonds between oxygen atoms from the guluronic monomers (G-blocks) and the cation. Particularly, the oxygen atoms of two G-blocks of two different polymer chains form a hydrophilic space where the cation can be introduced, generating a 3D network. The model that describes this interaction is known as the “egg-box” model [17]. The strength of these hydrogels can be influenced by the G/M ratio, as it affects the formation of “egg-box” structures [59,60].

**Figure 2:**
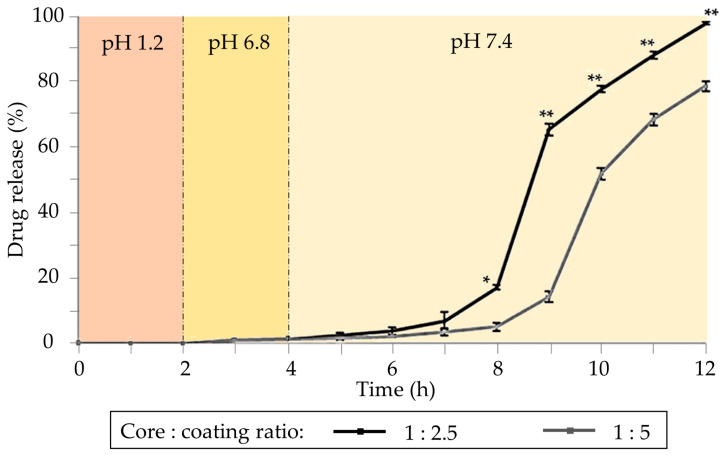
Cumulative drug release from coated formulations. The core is based on drug/alginate (1:6 weight ratio) coated with solutions of Eudragit^®^ S-100 with different concentrations (2.5% and 5% *w*/*v*). Notation: * Statistically significant difference at *p* < 0.001. ** Statistically significant difference at *p* < 0.0001 from 1:5 sample as determined by Student’s *t*-test. Image adapted from [74].

Different alginate gel formulations were developed to target the colonic tissue. Core–shell particles consisting of a core of alginate of varying polymer concentrations, loaded with sodium naproxen, and coated with Eudragit S100 were developed by the emulsification method [74]. The purpose of the Eudragit S100 coating was to safeguard the alginate in the stomach and duodenum, ensuring the release of naproxen in the colonic area. In vitro release tests in three different solution media (pH 1.2, 6.8, and 7.4) revealed variations in drug release rates among formulations (Figure 2). Complete release was achieved in all formulations, although a reduced release rate was observed as the concentration of Eudragit^®^ S-100 increased. Formulations effectively retain drug content within the acidic solution at pH 1.2, exhibiting minimal release as pH gradually rises to 6.8. The majority of drug release occurs only after the pH surpasses 7, requiring a minimum of 2 h of lag time to initiate the drug release.

Hybrid calcium alginate (CA) and carboxymethyl cellulose (CMC) beads were developed to modulate the release of 5-fluorouracil in the GIT [75]. Dry beads were produced by dropping mixtures of Alg (1.8% *w*/*v*) and CMC (0%, 0.5%, or 1% *w*/*v*) onto CaCl_2_ solutions (2% *w*/*v*), followed by an aging process involving a mixture of glutaraldehyde, ethanol (EtOH), and hydrochloric acid (HCl). These beads effectively loaded 5-fluorouracil through swelling and exhibited remarkable control of the drug release in an acidic medium (pH 1). At pH 7.4 and 6.8, the formulations experienced a burst release for the first hour, followed by sustained drug release lasting for 100 h. The addition of enzymes to the dissolution media promoted drug release after the first 24 h. Higher CMC concentrations in the formulations resulted in higher drug loadings and slower drug releases. Finally, these formulations released most of their drug content in the simulated colonic fluids.

### 3.2. Chitosan (CS)

Chitosan is the only semisynthetic cationic polysaccharide (Table 2) [47,59]. There are several varieties of chitosan of different molecular weights (MW) and deacetylation degrees (DD) ranging between 70 and 98%, depending on the specific alkaline treatment applied to the chitin [59,76]. CS has versatile applications in tissue engineering, wound healing, drug products (small molecules, DNA, and peptides), vaccines, cosmetics, and cancer diagnostics [61]. Its mucoadhesive properties make it an ideal candidate for colonic drug delivery, facilitating ionic interaction with the mucus wall (negatively charged) due to its positive charge [77]. In addition, CS acts as a permeation enhancer for transdermal drug delivery [78,79].

Nanoparticles (NPs) based on CS, or thiolate–chitosan, and alginate were prepared by the ionotropic gelation method [80]. These NPs were designed for the treatment of colorectal cancer and loaded with α-mangostin, an active compound with anti-inflammatory and antioxidative properties. Genipin was used as a crosslinking agent. In some formulations, Eudragit^®^ L-100 was added dropwise to the preparation solution to coat the NPs. The formulations crosslinked with genipin reduced the burst release to pH 1.2, whereas the Eudragit coating favored a controlled drug release at pH values over 1.2 and especially at pH 6.8 (50% drug released after 8 h). Additionally, these nanoparticles exhibited promising mucoadhesive properties, making them potential candidates for the treatment of colonic diseases.

### 3.3. Pectin

Pectin is an anionic heteropolysaccharide, also proposed for preparing colonic drug delivery systems as tablets, beads, pellets, or microparticles. It stands out for its resistance to degradation by intestinal enzymes while being susceptible to breakdown by colonic microflora [14,59]. The gelation properties of pectin and the behavior of pectin gels in gastrointestinal fluids depend on their degrees of amidation (DA) and esterification (DE). Low ester pectins (DE < 50%) can gel in the presence of calcium cations, while high ester pectins (DE > 50%) require acidic conditions and additional sugars to reach gelation. Similarly, the amidated groups within the pectin structure can enhance gelation in conjunction with calcium ions [14,81].

Core–shell polysaccharide microparticles containing betamethasone were prepared by coaxial prilling as a colonic formulation [82]. Amidated low-methoxy pectin was used as the core to preserve the drug, and alginate crosslinked with zinc was selected as the coating material. This type of coating exhibits high resistance against acid environments but degrades in the pH conditions of the small intestine. Once the colon was reached, the formulation could release betamethasone due to the degradation of pectin.

**Figure 3:**
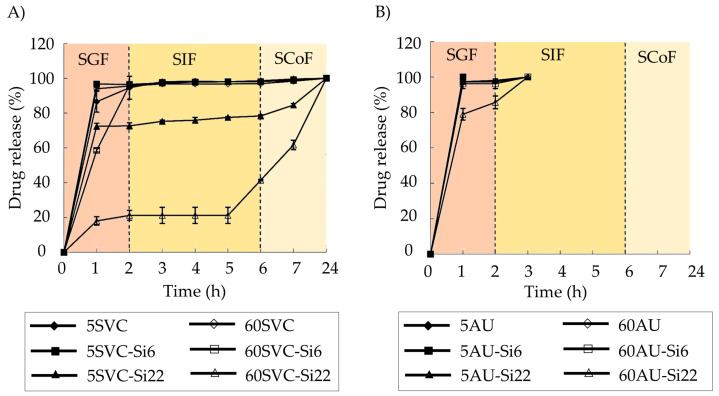
Mesalazine release profiles in the simulated digestive fluids for calcium pectin–silica and calcium pectinate gel beads based on the SVC (**A**) and AU701 (**B**) pectins. The formulations with high silica concentrations (22.2 mg/mL, Si22) and extended crosslinking times (60 min, 60) demonstrated the most suitable drug release profiles for colonic drug delivery applications. Image adapted from [83].

Pectin–silica beads containing mesalazine for IBD treatment were prepared [83]. Various pectin sources (*Silene vulgaris callus* pectin (SVC), *Lemna minor callus* pectin (LMC), and commercial apple pectin (AU)), crosslinking times (5 and 60 min) using 0.34 M CaCl_2_, and silica concentrations (0, 6.4, and 22.2 mg/mL) were used. These beads, of an average size of 1 mm, exhibited a colon-specific release profile of mesalazine, facilitated by a thin silica layer on their surface. Interestingly, this coating is formed spontaneously during the crosslinking process. The best-sustained release profiles were observed for those colonic formulations based on SLV and LMC pectins, in combination with the higher concentration of silica and 60 min of the aging process (Figure 3). This can be attributed to lower methyl-esterified groups in LMC and SVC pectins compared to the AU variety, enhancing their resistance to low pHs.

### 3.4. Cellulose

Cellulose is the most abundant homopolysaccharide on earth and has been widely used as an excipient in pharmaceutical formulations. Its modification by substitution of hydroxyl groups of the glucose units to include ether groups (methylcellulose, ethylcellulose, carboxymethylcellulose, hydroxyethylcellulose) or ester groups (acetate, nitrate, or sulfate groups) gives the molecule great variations in solubility, chemical structure, or gelation properties, opening possibilities for new applications in the biomedical field [62].

Coated cellulose-based pellets designed for the treatment of IBD were prepared using extrusion/spheronization [84]. Wet mixtures comprising microcrystalline cellulose (MCC), Carbopol 940 (CP940), high-substitute hydroxypropyl cellulose (H-HPC), sodium chloride (10.68% *w*/*w*), and specific amounts of either cyclosporine (2% *w*/*w*) or curcumin (4% *w*/*w*) were extruded, spheronized, and dried. Subsequently, pellets were coated with Eudragit^®^ S100 using a fluidized bed coater. The most effective formulation, exhibiting superior bioadhesive properties, consisted of 71.82% *w*/*w* of MCC, 5.75% *w*/*w* of CP940, and 5.75% *w*/*w* of H-HPC. Dissolution release profiles revealed that the most suitable pellets for colonic drug delivery were those coated with 20% weight gain relative to the initial formulation. These coated pellets maintained all drug content at pH 1.2 and promoted a constant drug release for 22 h in the intestine.

### 3.5. Starch

Starch is a polysaccharide widely used in the pharmaceutical industry as a disintegrant or binder in solid dosage forms [64]. Some varieties of resistant starch have been postulated as excipients for oral dosage forms for colonic drug delivery. They are obtained by retrogradation, a type of crystalline reorganization resulting from their heating in solution at high temperatures and subsequent cooling. Retrograded starches have limited hydration properties, making them less accessible to human enzymes and, therefore, less digestible than other starch varieties [47].

**Figure 4:**
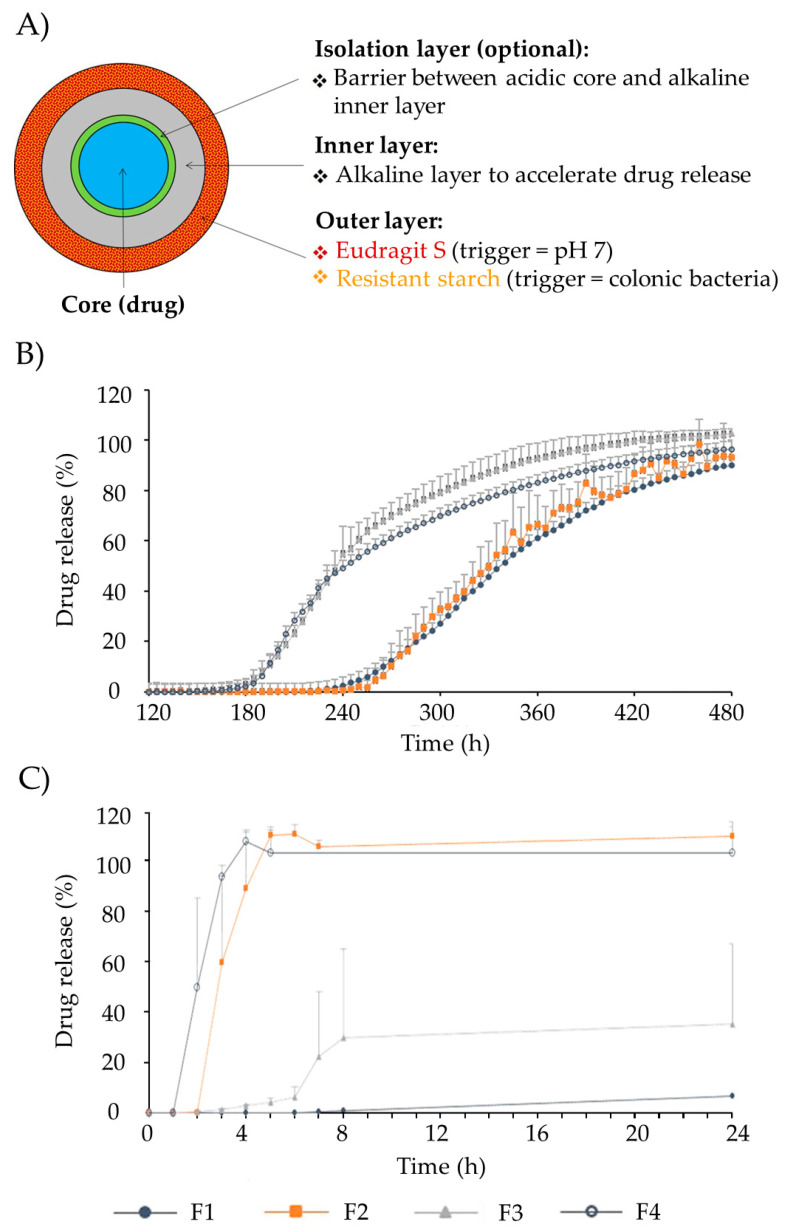
(**A**) Representation of the different layers that comprise the OPTICORE™ drug tablet system. (**B**) Effect of the starch-Eudragit outer layer on drug release patterns from several formulations, as follows: (F1) Single layer Eudragit^®^ S coating; (F2) Phloral layer (resistant starch and Eudragit S, without alkaline layer); (F3) Inner layer neutralized Eudragit^®^ S and outer layer Eudragit^®^ S; (F4) Outer layer Phloral™ (OPTICORE™) in Krebs buffer solution (pH 7.4). (**C**) Effect of the starch-Eudragit outer layer in drug release from formulations (mentioned in 7.B) in fecal human slurry (pH 6.8). Image adapted from [85].

OPTICORE™ is a multilayer technology based on resistant starches and Eudragit^®^ S coating, especially designed to target the colonic area [85]. Resistant starches allow drug release triggered by colonic enzymatic activity and Eudragit^®^ S by the pH variation in the medium (Figure 4A). An additional alkaline layer accelerates drug release. OPTICORE™ system (F14) starts the release 1 h after achieving simulated colonic fluids, while in conventional formulations based on Eudragit^®^ S (F1), the drug release starts 2 h after reaching the colon, increasing the possibility of failure of the treatment (Figure 4B). Also, when the formulations are prepared without starch and evaluated in a human fecal slurry (F2 had the outer layer of the OPTICORE™ formulation without the alkaline layer, and F3 was composed of an alkaline layer and an Eudragit^®^ S layer), drug release is achieved only with formulations with resistant starch in their composition (Figure 4C). This was due to this fecal medium having a pH of 6.8, representative of the pH medium of the large intestine, which is insufficient to degrade the Eudragit^®^ S layer. Consequently, the formulation that guarantees a sustained release with a complete release independently of the pH conditions is based on OPTICORE™.

### 3.6. Konjac Glucomannan (KGM)

Konjac glucomannan is a non-ionic polysaccharide widely used in traditional Asian medicine due to its laxative, anti-obesity, antidiabetic, and anti-inflammatory properties. Upon hydration, KGM has a unique capacity to absorb water and expand significantly, improving peristaltic movements. Furthermore, hydrated KGM gel prevents the absorption of fatty acids and different sugars in the GIT, reducing hyperglycemic peaks [66]. KGM has been used for different biotechnological and chemical applications within the biomedical field, like tissue engineering [86], cosmetics [87], or the encapsulation of bioactive compounds [88].

**Figure 5:**
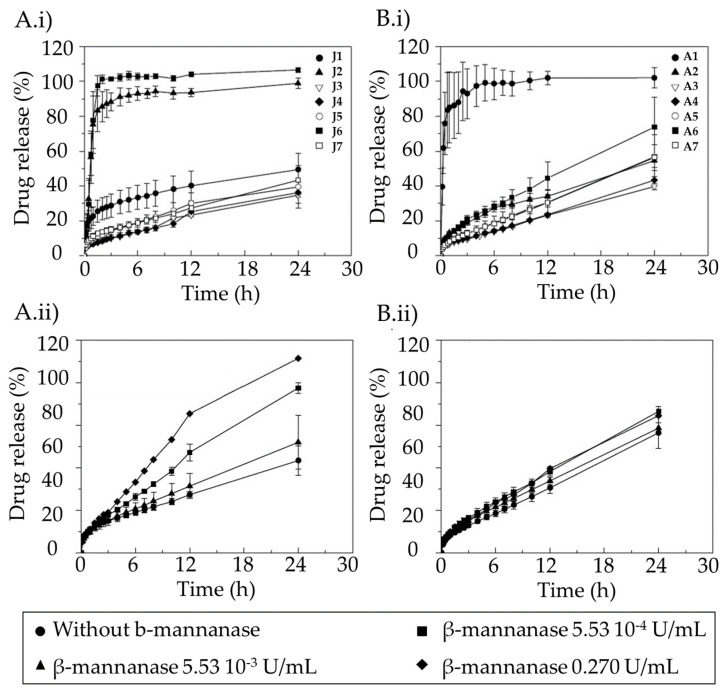
Dissolution profiles of (**A.i**,**B.i**) diltiazem into the simulated intestinal fluid (SIF) without β-mannanase and release profiles of the drug from formulations prepared with Japanese KGM (**A.i**,**A.ii**) and American KGM (**B.i**,**B.ii**), and (**A.ii**,**B.ii**) containing various concentrations of β-mannanase for formulations J7 (**A.i**,**A.ii**) and A7 (**B.i**,**B.ii**). Image extracted from [89].

Hydrophilic matrices based on mixtures of KGM, xanthan gum, and sucrose have shown adequate properties to achieve colonic drug delivery (Figure 5) [89]. These types of matrices controlled the release of highly soluble drugs, such as diltiazem, for long periods of time. KGMs from different origins have shown variations in the control of diltiazem release (Figure 5). Both matrices of Japanese KGM (Figure 5A.i) and American KGM (Figure 5B.i) under certain proportions in the formulation allowed the control drug release. The addition of β-mannanase to the dissolution medium containing J7 or A7 formulations (Figure 5A.ii,B.ii) accelerated the release of the drug, especially for the Japanese KGM (Figure 5A.ii), which has demonstrated the specificity of the formulation for the colonic area.

### 3.7. Other Polysaccharides

Other polysaccharides of marine (agar), microbial (xanthan gum), and plant (guar gum and locust bean gum) origins have also been studied for colonic administration purposes [90].

Agar has the remarkable ability to create strong gels when an aqueous suspension is prepared and subjected to a heating process between 65 and 100 °C, followed by a cooling step (4–8 °C) to achieve complete and homogeneous gelation [69,91]. This technique has been widely applied in the formulation of various hydrogels designed for delivery systems [92]. These structures show exceptional resistance to disintegration in SIF, and their adequate coating with chitosan allows probiotics to reach the colonic area.

Xanthan gum has a polyanionic composition and is soluble in hot and cold water [59,69]. Its combination with other polysaccharides, such as locust bean gum or agarose, and its processing by autoclaving allow the formation of hydrogels with diverse mechanical properties that have been studied as delivery systems [69,93].

Guar gum is a polysaccharide extracted from the seeds of *Cyamopsis tetragonolobus*, known for its high stability against gastrointestinal fluids, making it an ideal candidate for use in coatings or as matrix systems [47]. Colonic administration systems employing guar gum can be prepared by compression [94,95] or by employing water-in-oil emulsification with a simultaneous incorporation of acrylic acids [96] or succinic anhydride [97].

New derivatives have also been produced from existing polysaccharides. An example is dextrins, which are produced by partial acidic or enzymatic hydrolysis of starch, resulting in low-molecular-weight amylose or amylopectin chains [98]. They can be chemically modified to create pH-responsive coatings for drug delivery systems, thus allowing controlled release in specific environments [99].

## 4. Polysaccharide-Based Aerogels as Dry Carriers for Colonic Delivery

There is a paucity of information on polysaccharide-based aerogels for oral drug administration, with even more limited data on their colonic administration applications. However, they offer several advantages, such as their economical and environmentally friendly preparation, which makes them interesting materials for this purpose. Their mesoporous structure allows them to keep the active compounds in the amorphous state, thereby enhancing the drug solubility characteristics beyond what conventional oral pharmaceutical forms can achieve [20]. For colonic administration, polysaccharide-based aerogels can also be designed as matrix systems whose enzyme-resistant properties serve to preserve the drug until it reaches the colon [47]. Alternatively, they can be developed as release-modulated coated systems, with drug release controlled by the disintegration induced by the colonic flora [47]. These benefits enable lower administration doses to achieve the desired therapeutic effects, reducing side effects and treatment costs. Furthermore, the specific drug delivery in the colonic area should allow the local treatment of pathologies such as IBD or colon cancer [100].

The subsequent sections provide an overview of the available information regarding the methodologies for preparing polysaccharide-based aerogels and loading them with drugs of different solubility characteristics. Furthermore, these sections will explore the ongoing advances in the design of dosage systems with aerogels, whether monolithic or multiparticulate. Special mention will be given to the types of drug delivery achieved and their potential adaptation for colonic applications. Finally, the topic of aerogel coating will be addressed as an additional step to successfully reach delivery in the colonic region.

### 4.1. Polysaccharide-Based Aerogel Preparation

**Figure 6:**
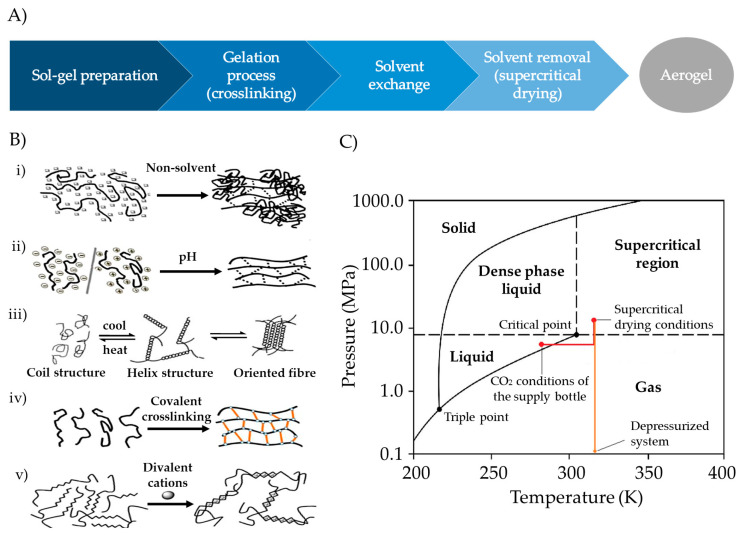
(**A**) Stepwise procedure for polysaccharide-based aerogel preparation. (**B**) Crosslinking techniques for gelation: (i) NIPS, (ii) pH-induced gelation, (iii) temperature-induced gelation, (iv) chemical gelation, and (v) ionotropic gelation. Image adapted from [17]. (**C**) CO_2_ phase diagram used for the SCF drying process. CO_2_ is continuously pumped and heated until SCF drying temperature and pressure conditions are achieved. After drying, depressurization must be carried out at a specific temperature and flow rate until the atmospheric pressure is reached. Image adapted from [107].

The preparation of aerogels typically follows a stepwise procedure, encompassing sol–gel preparation, gel crosslinking, solvent exchange, and solvent removal (Figure 6A) [101]. Polysaccharide-based sol–gels are prepared by dispersing solid polymers in an aqueous medium. Subsequently, crosslinking can be induced by employing methods such as pH-induced gelation, non-solvent-induced phase separation (NIPS), temperature-induced gelation, covalent crosslinking, or ionotropic gelation (Figure 6B), depending on the functional groups in the polymer molecule structure [17,20,102]. Gelation significantly influences the textural properties of the aerogel, determined by the union between the molecular chains of the polysaccharides [17,18,20,103].

After preparing a gel, different techniques can be used for drying, but many of them struggle to counteract the capillary forces that may lead to the collapse of the mesoporous structure within the gel. Among them, vacuum drying or oven drying results in materials known as xerogels, while freeze-drying typically yields cryogels [18,104]. Alternatively, aerogels are usually obtained by SCF drying with supercritical CO_2_ (scCO_2_). Due to the reduced capacity of CO_2_ to solubilize water, SCF drying with scCO_2_ requires an additional step of replacing the water in the hydrogel with alcohol (solvent exchange), resulting in an alcogel [17,20,81]. Next, ethanol is removed from the alcogels in a high-pressure autoclave using scCO_2_ (Figure 6C) [105,106,107]. scCO_2_ demonstrates superior permeation within porous structures compared to liquids and shows greater solvation capacity for dissolving substances than gases. Consequently, they can effectively eliminate the solvent from gels, avoiding capillary forces and preserving the mesoporous structures of the original wet material [18,105,108].

### 4.2. Drug Loading within Aerogels Impregnation Technique

**Figure 7:**
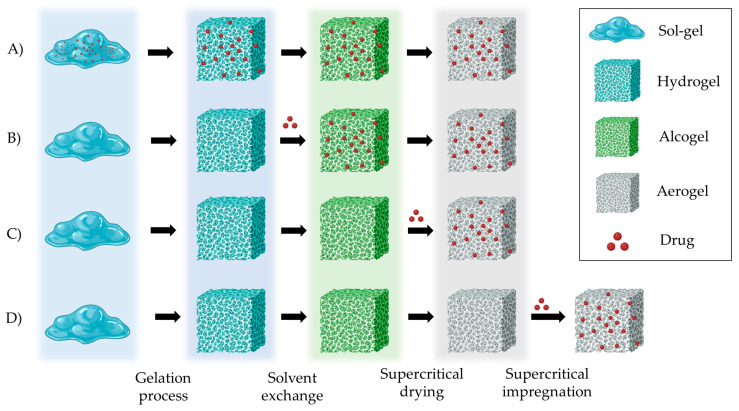
Strategies for preparing drug-loaded aerogels. (**A**) Incorporating the drug into the gel solution; (**B**) Adding the drug into the solvent for loading in the gels by solvent diffusion; (**C**) Adding the drug during the SCF drying process; (**D**) Loading the drug into the pre-prepared aerogels by impregnation technique. Notation: Red dots represent the drug that is loaded in the aerogel carrier.

Drug loading into the aerogel structures can be carried out at different steps of the production process: into the sol–gel mixture, during the solvent exchange, or during the SCF drying (Figure 7). The solubility of the active compound is a key parameter in selecting the loading strategy and determining the drug loading efficiency. Hydrophilic drugs are good candidates to be loaded into the gel solution prior to the crosslinking process. Hydrophobic drugs can be loaded during solvent exchange or SCF drying. Moreover, drug loading can be performed after SCF drying by adsorption–precipitation method (supercritical impregnation) [20,109].

**Figure 8:**
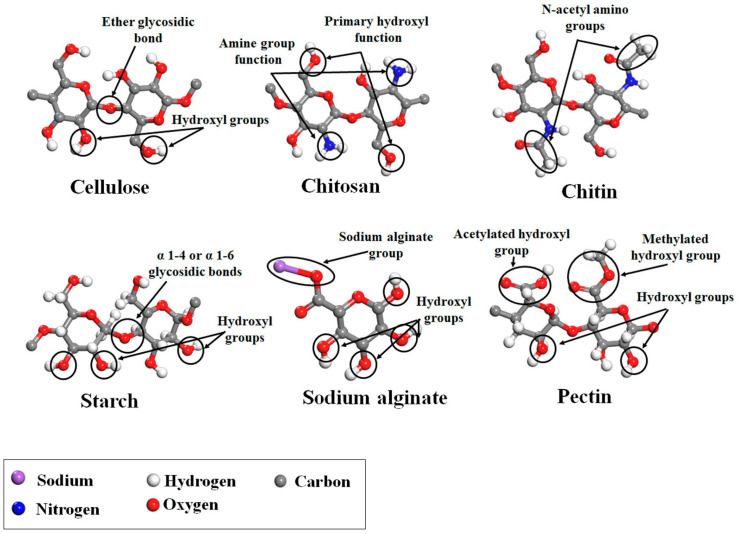
Main monomer functional groups of polysaccharide aerogels. Image adapted from [110].

The supercritical impregnation process is typically carried out by conditioning the aerogel and the active substance in permeable cartridges in a high-pressure autoclave. Injection of supercritical CO_2_ at a suitable pressure and temperature for a certain time (typically several hours) will achieve equilibrium between the drug-saturated scCO_2_ and the internal porous matrix of the aerogels. Controlled depressurization is necessary to return to atmospheric conditions and eliminate CO_2_ [20,109]. A successful impregnation process requires a careful definition of various factors. Initially, it is crucial to ensure that the drug is soluble in scCO_2_. Consequently, potential alterations in temperature and pressure values or the addition of cosolvents must be considered, as they can significantly influence the solubility of the drug in the supercritical medium [109]. The depressurization rate can directly influence the amount of drug retained in the matrix structure and its crystalline state. The aerogel composition and its microstructure influence the amount of drug adsorbed on the porous surface [20,109,110]. The type and density of functional groups in the matrix porous structure determine the affinity of the drug molecule for the skeletal structure of the aerogel and, therefore, the impregnation process [110]. The functional groups of the primary monomers in biopolymer aerogels, which directly influence their impregnation capability, are illustrated in Figure 8 [110].

The effect of the abovementioned factors is exemplified in the results from polysaccharide aerogels of pectin, alginate, and starch produced through the emulsion–gelation method and SCF drying [103]. The loading of these aerogels was carried out by impregnation at 180 bar (40 °C for ketoprofen and 55 °C for benzoic acid) for 24 h. Aerogels with a greater surface area exhibited more efficient drug loading. The drug loading efficiency was also influenced by the type and density of functional groups (hydroxyl, carboxyl, and amino) in the skeletal structure of the aerogels, in alignment with the molecular structure of benzoic acid and ketoprofen. Specifically, the molecular structure of the starch-based aerogels promoted higher ketoprofen loading. This can be attributed to starch’s higher concentration of hydroxyl groups in comparison to other polymers and its lack of acid groups. Lastly, the molecular weight of the drugs can also dictate the loading efficiency, with smaller molecules being more favorable for impregnation.

### 4.3. Polysaccharide-Based Aerogel Dosage Systems: Production and Release Properties

The combination of various aerogel production techniques with different gelation and drug loading methods yields aerogel dosage systems of varying shapes and distinct behaviors [103]. The shape, together with the aerogel composition and the drug loading technique, have a strong influence on the drug release profile of the formulations (Table 3).

**Figure 9:**
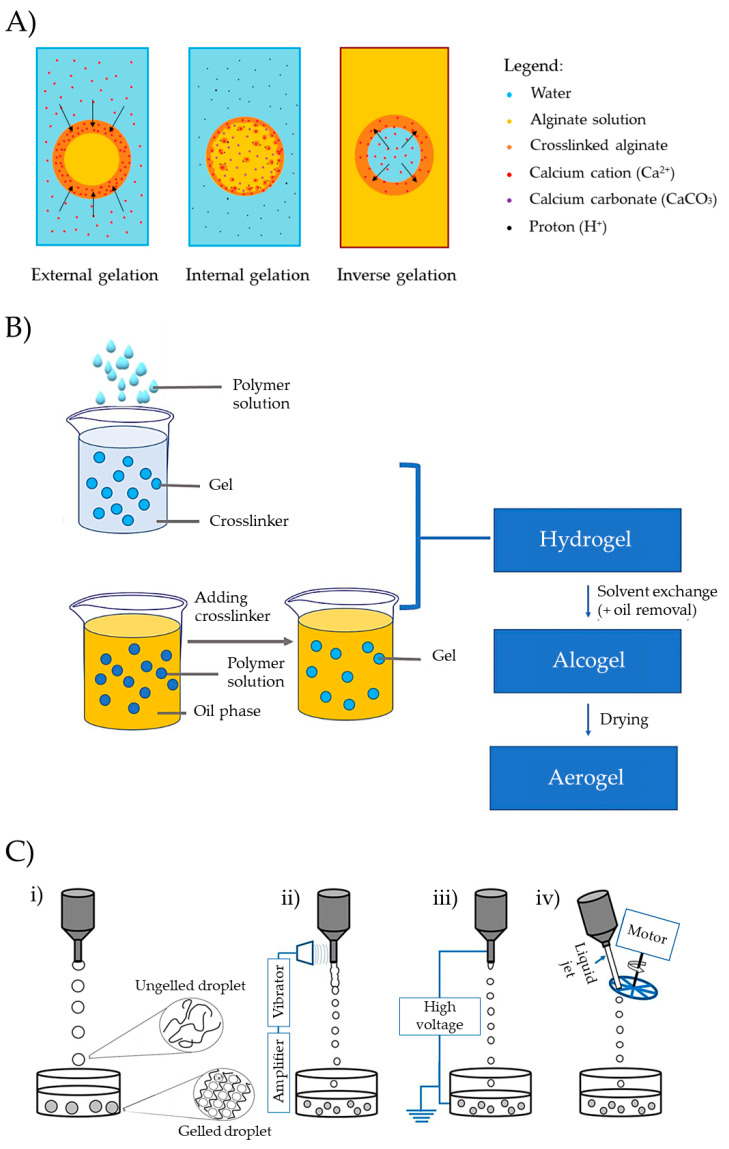
(**A**) Representation of external, internal, or inverse gelation. This figure illustrates the gelation process of alginate with Ca^2+^ as an example, but the underlying concept is applicable to various polymers and crosslinking agents. The arrows indicate the direction of the crosslinking front. (**B**) Preparation of aerogel microparticles by internal gelation and external gelation. Image extracted from [100]. (**C**) Dripping gelation modalities: (i) conventional, (ii) vibrating, (iii) electrostatic, and (iv) jet-cutting dripping methods. Image extracted from [17].

Depending on the location of the crosslinking agent (Figure 9A), gelation can be classified as external, internal, or inverse [17]. External gelation involves introducing the polymer solution into a bath containing the gelation promoter (e.g., salts and organic solvents) [125]. Alternatively, a water-in-oil (W/O) emulsion of the polymer can be prepared, followed by the dropwise addition of a crosslinking agent into the emulsion to induce polymer gelation [126]. Internal gelation involves adding the crosslinking agent in an insoluble state into the polymer solution and then dropping this mixture in a bath capable of solubilizing the crosslinking agent, thereby promoting the gelation of the polymer. This procedure is especially convenient for the gelation of Alg. It involves adding calcium carbonate (CaCO_3_) to an Alg solution and then dropping the Alg in an acidic bath. This promotes the dissolution of calcium and the gelation of Alg. Alternatively, it is also feasible to introduce an acid solution dropwise into an emulsion containing Alg and CaCO_3_ dispersed within Alg [127]. Finally, in the process of inverse gelation, the crosslinking agent (e.g., in a water solution) is dropped into a polymer solution, promoting the gelation from the innermost droplet and progressing outward [128].

Molds of various shapes, such as cylinders or spheres, can be used to prepare monolithic forms. Examples of monolithic shapes prepared from starch (Entry 1) [111], pectin (Entry 2) [112], or kappa-carrageenan (Entries 7 and 10) [117,121] are presented in Table 3. Cylinders can also be produced through extrusion, and other shapes can be achieved using 3D printers [103]. Various technologies, such as emulsification, drip-gelation, or prilling, allow the development of multiparticle aerogel systems that can load drugs in an amorphous state, enabling controlled release. These technologies offer promising prospects for improving absorption and stability [20]. The choice of technique significantly influences the size and morphology of the gel particles, with emulsion–gelation and dripping being among the most commonly employed methods (Figure 9B) [23,103]. Emulsion gelation techniques create solid particulate aerogels by forming a W/O emulsion with polysaccharides in the aqueous phase and liquid paraffin in the oil phase. Emulsion droplets ranging from 1 to 3000 µm in size are crosslinked, washed, and dried to successfully produce solid multiparticulate aerogels. This method has been widely explored for polysaccharide-based aerogels of alginate, starch, and pectin (Entry 13) [22] and MCC (Entry 3) [113].

Dripping a polymer solution into a gelation bath is also a versatile technique to obtain multiparticulate systems. The bath composition dictates the gelation process (ionic crosslinking, coagulation), while the dripping method (conventional, vibrating, electrostatic, or mechanical cutting) influences particle characteristics. The conventional method relies on factors such as solution viscosity, surface tension, nozzle diameter, and gravity forces to form spherical structures in the air with specific dimensions, followed by gelation in the bath. Alternative modalities employ different physical principles to break the preformed drops within the nozzle, enhancing efficiency or reducing particle size or shape (Figure 9C) [17,103,129]. The scale-up of the dripping gelation technique can be carried out by using multiple nozzles to drop the formulations simultaneously into the bath. This method consists of installing a rack of nozzles of the same geometry over a crosslinking bath and keeping the same distance between the bath and the tip of the nozzles [130].

Multiparticulate aerogels have been produced with pectin by mechanical-cutting or jet-cutting dripping technique (Entry 5) [115], Alg by vibrating dripping technique (Entry 15) [124], or pectin or alginate aerogels by conventional dripping technique (Entries 4 [114] and 14 [123]) (Table 3).

**Figure 10:**
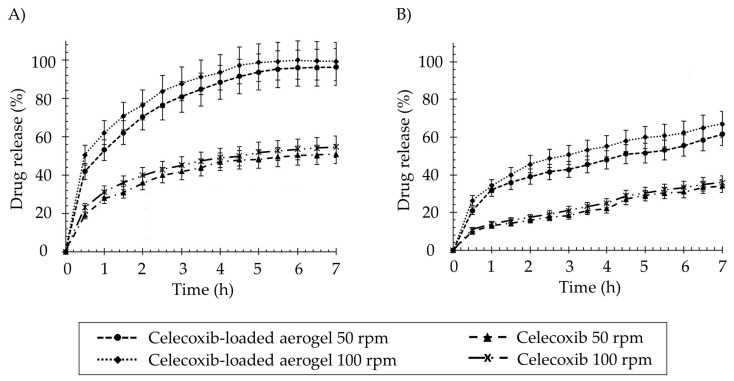
Release profiles of celecoxib (raw material) and celecoxib-loaded starch aerogels in different dissolution media (**A**) SGF medium and (**B**) SIF medium and paddle speeds (50 and 100 rpm). Significant enhancements can be observed in the cumulative release percentage of celecoxib from the starch aerogel systems compared to the raw material under all tested conditions. Image extracted from [111].

Polysaccharide-based aerogel systems can be used to improve the solubilization characteristics of poorly soluble drugs. For example, starch aerogel monoliths were prepared by dissolving the starch powder in water at 14.1% *w*/*v*, heating at 117 °C, and cooling to promote the retrogradation process (Entry 1, Table 3). Afterward, a solvent exchange and subsequent SCF drying were carried out. They were loaded with celecoxib during the solvent exchange. They have shown faster drug release compared to the raw material as supplied in both SIF (pH 7.4) and SGF (pH 1.2) at any of the paddle speeds studied (50 and 100 rpm) (Figure 10) [111]. This is undoubtedly related to the large dissolution surface area of the porous structures of the aerogel. The drug release profiles fit the Korsmeyer–Peppas model, demonstrating the diffusion–erosion mechanism. The Fickian diffusion of celecoxib is complemented by the degradation of the aerogel matrix structure.

**Figure 11:**
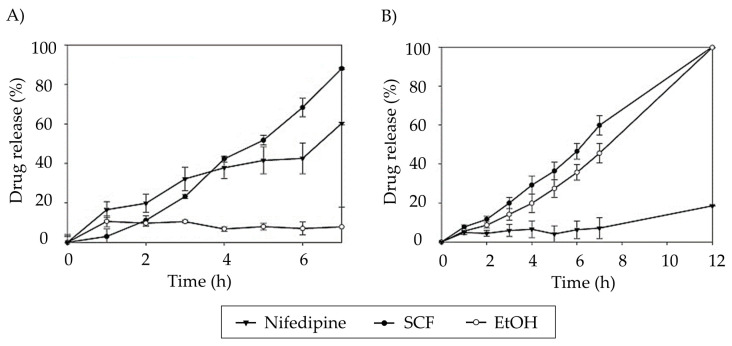
Comparison of nifedipine release profiles from its raw crystalline state and pectin aerogels loaded via two distinct methods, solvent exchange in ethanol (EtOH) and in supercritical fluids (SCF), in two dissolution media: (**A**) SGF (pH 1.2) and (**B**) PBS (pH 7.4). Image extracted from [112].

Monolithic aerogels with high methoxyl pectin content have also been proposed to improve the oral bioavailability of nifedipine (Entry 2, Table 3) [112]. Aqueous pectin solutions (1, 2, and 4% *w*/*v*) were molded, gelled in 10% v/v EtOH, solvent exchanged (absolute EtOH), and scCO_2_ dried. Nifedipine loading occurred during solvent exchange (3 h in a drug-saturated solution) or impregnation after drying (200 bar and 60 °C for 24 h). Incorporating nifedipine into pectin aerogels has not significantly enhanced its release in SGF; in fact, it notably delays the release, particularly for aerogels loaded during the solvent exchange (Figure 11A). However, the formulation of nifedipine in pectin aerogels expedites drug release in neutral-basic media (Figure 11B), especially when loaded through SCF impregnation. In this case, the profiles fit the Korsmeyer–Peppas model, indicating a mechanism of erosion-diffusion for those aerogels in PBS medium. 

Polysaccharide-based aerogels can also be engineered to control drug release within the GIT. For instance, cellulose aerogels loaded with acetaminophen were prepared using the emulsion–gelation method to address this goal (Entry 3, Table 3) [113]. Cellulose was first pretreated with dimethylformamide at 100 °C, dissolved in 1-butyl-3-methylimidazolium chloride (an ionic liquid), and emulsified in a cyclohexane solution with Hypermer 1599™ and Tween^®^ 80 as additives. The resulting emulsion was scCO_2_-dried. The acetaminophen loading was carried out during the solvent exchange step (absolute EtOH). Cellulose aerogels (3% *w*/*v*) effectively controlled acetaminophen release in PBS (pH 7.4) in comparison with acetaminophen crystals. Release profiles followed first-order kinetics, suggesting diffusion in water as the main release mechanism, with no observable carrier degradation except for initial shrinkage in the first few minutes. These systems have the potential for targeting colon drug delivery, where microorganisms can degrade cellulose structures and promote drug release.

**Figure 12:**
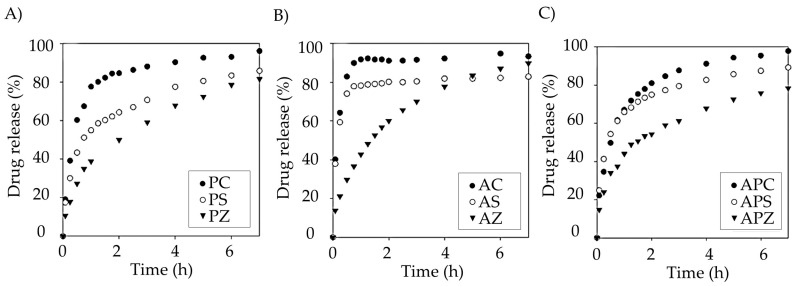
Drug release profiles in PBS medium from different polysaccharide-based aerogels: (**A**) pectin, (**B**) alginate, and (**C**) alginate–pectin (1:1), ionically crosslinked with divalent calcium ions (PC^+^), strontium ions (PS), and zinc ions (PZ). The figure illustrates the differences between aerogels produced with different compositions as well as the substantial effect of the type of crosslinking agent on drug release. Image extracted from [114].

Controlled diclofenac release has also been achieved from Alg, low-methoxy pectin, and their mixtures (1:1) aerogels prepared through the dripping method (Entry 4, Table 3) [114]. Solutions of polysaccharide (2% *w*/*v*) containing diclofenac (1% *w*/*v*) were dropped, crosslinked with Ca^2+^, Zn^2+^, or Sr^2+^ (2% *w*/*v*), cured for 24 h, and supercritically dried (100 bar and 40 °C) to produce the aerogels. All aerogels were stable in SGF over a period of 7 h and started to swell when in contact with PBS. Figure 12 presents the diclofenac release profiles in a PBS medium (pH 6.8), showing a strong dependence on the polysaccharide composition and the crosslinking agent. Calcium-crosslinked aerogels provided the highest surface areas, and zinc crosslinked the lowest. As a result, pectin aerogels crosslinked with zinc exhibit slower drug release, making them more promising as controlled delivery systems.

### 4.4. Coating of Polysaccharide-Based Aerogels for Colonic Applications

Coated polysaccharide-based aerogels are regarded as a valuable tool to develop formulations for colonic administration. This approach is expected to reduce drug losses in the upper regions of the GIT while boosting the local drug dose in the colon. Despite existing examples of aerogel coating in the literature (Table 4), none of them have demonstrated the creation of a colonic delivery system. Nevertheless, the conducted research can serve as the basis for designing effective colonic formulations, as coating has the potential to impact mechanical properties, slow water uptake, and modify the drug release mechanism.

**Figure 13:**
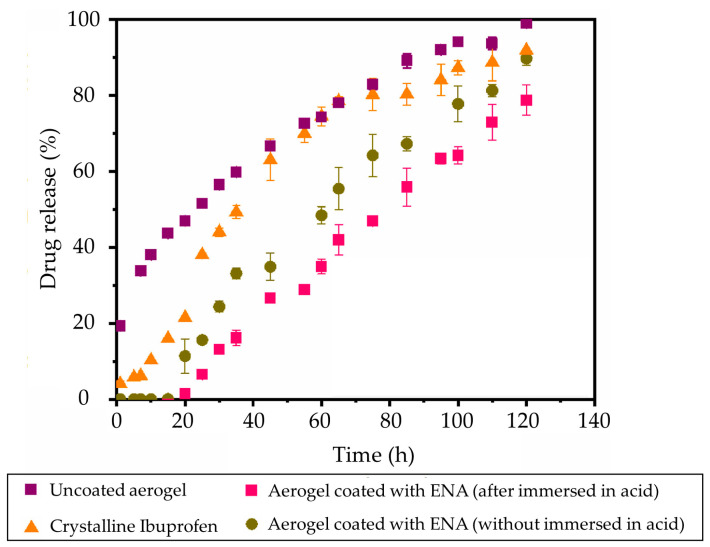
Release profiles of ibuprofen from a crystalline form and coated and uncoated aerogels in PBS medium (pH 7.4). Image extracted from [131].

A method used for coating polysaccharide-based aerogels is the fluidized bed technique that has been used to modify drug release profiles [131]. For instance, Alg aerogels were produced through dripping, ionic crosslinking (Ca^2+^), and SCF drying. These aerogels were loaded with ibuprofen through impregnation (at 200 bar, 40 °C, 3 h), and a Wurster fluidized bed was used to apply a coating with an aqueous dispersion of methacrylic acid-ethyl acrylate (“Aquarius™ Control ENA”, ENA) at 60 °C. Under these conditions, the coating process did not alter the porous structure of the aerogel or modify the release of the drug. Under acidic conditions, the particles exhibited a well-preserved coating without drug release due to their enteric coat. Under basic conditions (PBS, pH 7.4), the coated particles initially retained the ibuprofen. The formation of microscopic pores in the coating after 15 min facilitated the controlled release of ibuprofen (Figure 13).

In another example, coated cellulose aerogels were prepared using spouted bed technology and evaluated for colonic purposes [132]. The particles were produced by jet-cutting dripping of cellulose dispersions into an alkaline aqueous medium, followed by drying and supercritical impregnation with vanillin (125 bar, 60 °C, 16 h). The particles were coated with ethanolic solutions of shellac (a natural resin) at different concentrations. The uncoated aerogels released 50% of the vanillin content during the first 20 min, while the coating with a volume/mass ratio of approximately 1 mL/g was able to retain 50% of the vanillin content for up to 90 min.

The coating can also modify the water uptake and mechanical properties of the aerogel. Hybrid Alg–starch aerogels with a cylindrical tablet shape were prepared in a fluidizer bed coater [133]. CaCO_3_ was dispersed into the polymer solution to promote the gelation process. Afterward, a solvent exchange to EtOH, followed by an SCF drying process, was carried out. For the coating of the aerogels, a fluidized bed coater was used to spray an aqueous Eudragit^®^ 30 D-55 solution at 30% *w*/*v*. Water uptake data for the coated aerogels exhibited minimal values at low pH (pH 1.2) and increased from pH 5.5 as the coating became permeable. At pH 7.4, water uptake was unconstrained.

Aerogel coatings can be produced through alternative methods such as cold plasma processes or immersion. When employing the cold plasma coating technique with perfluoro-acrylates (e.g., 1H,1H,2H,2H-perfluorooctyl acrylate), it becomes possible to effectively modulate the surface wettability of polysaccharide-based aerogels like Alg or cellulose, making them superhydrophobic. Furthermore, this deposition method offers flexibility and quick processing times [134].

The multi-step sol-gel process is a straightforward technique that has enabled the preparation of multilayer hydrogels and, therefore, aerogels. Alg beads at a concentration of 1.5% *w*/*v* were coated by immersing them in successive layers of alginate solution at 0.75% *w*/*v*. The wetted beads were then collected and subjected to gelation in a 0.2 M CaCl_2_ solution. This procedure allowed the incorporation of a water-soluble drug (nicotinic acid) either within the initial bead cores or within distinct layers. The subsequent solvent exchange and SCF drying steps enable the production of loaded aerogels, whose controlled drug release depends on the number of layers incorporated [135]. 

Coaxial prilling is another method that enables the production of coated aerogels with remarkable versatility. This innovative technique allows for the one-step preparation of core–shell hydrogels, which, upon SCF drying, results in the formation of core–shell aerogels [136]. As an example, spherical core–shell particles (mean diameter: 3.25 mm) were achieved by pumping an inner solution of 3.5% *w*/*v* pectin containing doxycycline (3.5% *w*/*w*) through a 0.4 mm internal nozzle while simultaneously employing an annular solution of 1.5–1.75% *w*/*v* aqueous Alg through a 0.6 mm outer nozzle. The crosslinking process occurred in a gelling bath (0.5 M CaCl_2_ dissolved in 96° EtOH). A vibration frequency of 350 Hz was maintained with the coaxial nozzle positioned 25 cm away from the crosslinking bath. After the core–shell beads were prepared, they were dried using scCO_2_ technology. The obtained particles had an encapsulation efficiency of up to 87%, and the sustained drug release was maintained for 48 h. 

The preceding examples clearly demonstrate the considerable potential of polysaccharide aerogels as effective oral drug delivery systems, particularly for colonic administration. Nevertheless, the existing body of research on aerogels tailored for colonic applications remains quite limited. Despite the extensive research dedicated to developing polysaccharide-based aerogels with Alg, cellulose, starch, or CS, there remains a critical need to address the gaps in understanding the ideal composition characteristics and operational conditions necessary for these systems to effectively reach the colonic area and optimize drug release for efficient treatments [20]. While certain strategies, such as pH-dependent polymer coatings, offer potential improvements in release profiles, none of the existing studies have conclusively demonstrated their specificity at the colonic level.

## 5. Future Trends in Research on Aerogels as Colonic Drug Delivery Systems

Undoubtedly, the rise in colonic pathologies, including colorectal cancer, Crohn’s disease, ulcerative colitis, diverticulitis, and irritable bowel syndrome, can be attributed to a combination of factors, such as diet, lifestyle, and various other determinants that the world, especially the more industrialized one, is currently experiencing. For example, the number of new cases of colorectal cancer in 2020 was higher than 1.9 million worldwide, according to the World Health Organization [137]. Also, the prevalence of IBD has increased significantly. Particularly in Western countries, Europe, and the United States, the prevalence is already between 0.5 and 0.75% [138,139]. Those diseases greatly impact patients’ quality of life, hinder their productivity [5,140,141], and pose an extraordinary burden on treatment costs for national health systems [142].

In this scenario, innovation in colonic drug delivery systems designed to target local treatment of these pathologies, facilitate the administration of NME (poor-soluble drugs, peptides, or proteins), or improve the effectiveness of existing drugs offers promising prospects.

According to the precedent sections, the possibility of engineered polysaccharide-based aerogels that can respond to pH, enzymes within the colon microbiota, and even specific molecules as reactive oxygen species (ROS) produced by the inflammatory cells involved in these pathologies is a reality, but also a path that remains relatively unexplored [20,143]. The selection of polysaccharides first determines their release, which can be any of those mentioned or even a combination of two of them, giving the aerogels great selectivity to target the colon.

The literature review has shown that the most successful strategy for polysaccharide aerogels to reach the colon while avoiding the early release of the drug is the formation of monolithic or multiparticulate systems coated with pH or enzymatic-resistant materials. Consequently, the development of core–shell formulations of aerogels presents the best prospects.

Work involving the development of coatings with synthetic polymers like Eudragit^®^ typically employs fluid bed coaters or spouted bed technology, which have shown excellent results in terms of maintenance of the aerogel structures and release profiles. These techniques are widely recognized and easily scalable. Nonetheless, there is potential for innovation in producing core gel particles with polysaccharide shells, offering interesting possibilities for combining various functionalities, such as mucoadhesion and responsiveness to pH or both pH and enzymatic responses.

Coated aerogels can also be prepared by immersing aerogel cores in diverse polysaccharide shells using coaxial nozzles and SCF drying. This method enables the formation of multiparticle aerogel systems with variable sizes and offers a range of release mechanisms, allowing for customized release rates [136]. Its versatility extends to the use of various polysaccharide combinations, high production rates, and straightforward scalability [144,145]. Despite the considerable potential of this technique to advance the development of colonic aerogels, a notable gap exists in effectively incorporating poorly soluble drugs or other types of NME at appropriate doses and demonstrating the influence of the system properties on their bioavailability.

The powder coating method for aerogels is an unexplored approach that entails mixing the final aerogel formulation with low-melting atomized materials. Upon heating, fusion and coalescence occur, creating a lasting coating on the particle surfaces. This coating will persist after cooling [146]. This technique is environmentally friendly, as it eliminates the use of organic solvents in coating solutions. 

Treatments for cancer or IBD frequently include antitumorals, anti-inflammatories, corticosteroids, immunomodulators, antibiotics, and monoclonal antibodies, some of which have poor bioavailability due to their low solubility properties [5]. The inclusion of these drugs in the amorphous state in stable and solid aerogel formulations should help overcome solubilization problems and enhance the efficacy of treatments [20]. Some of these active compounds have already been formulated into coated aerogels with the aim of colonic administration (Table 4).

There is a paucity of knowledge concerning the drug crystalline state within aerogels and its evolution upon storage, particularly in terms of maintaining stability in the amorphous state and solubility characteristics [20,109]. There is also no information in the literature about the mucoadhesive properties of the polysaccharide-based aerogel particles formulated for colonic delivery purposes. Additionally, drug release profiles are not fully explored. The impact of the microbiota on the drug-release process from these aerogels remains undocumented, and there is a lack of in vivo studies in the existing literature. 

These areas represent critical research frontiers that warrant further exploration and investigation within the field of polysaccharide-based aerogels as colonic delivery systems.

## Figures and Tables

**Table 2:** Main types of polysaccharides used to develop colonic formulations.

Polysaccharide	Structure	Composition	Sources	References
Alginate (Alg)	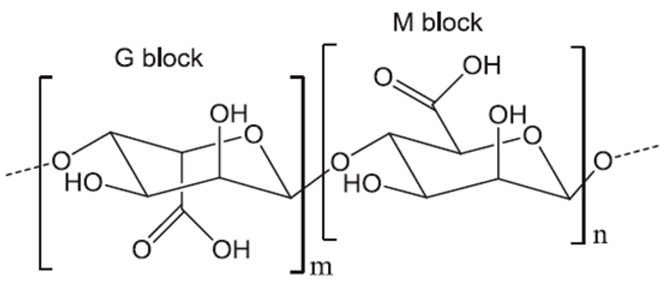	β-(1→4)-D-mannuronic acid (M) and α-(1→4)-D-guluronic acid (G) are linked by β-(1-4)-glycosidic bonds in different ratios, according to the source.	Marine brown algae and microorganisms	[59,60]
Chitosan (CS)	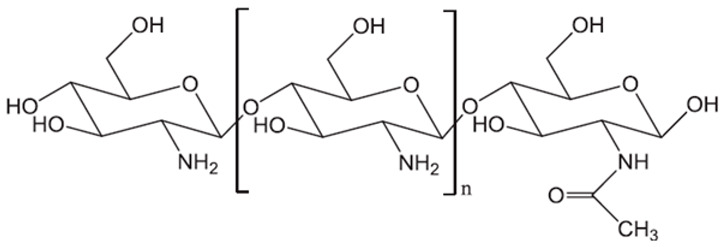	N-acetyl-2-amino-2-deoxy-d-glucopyranose (acetylated unit) and 2-amino 2-deoxyd-glucopyranose (deacetylated unit) are linked by β-(1→4)-glycosidic bonds. DD (70–98%) and viscosity in specific conditions (100–5000 mPa∙s).	Chitin deacetylation of exo- and endoskeletons of crustaceans, cephalopods, and insects	[59,61]
Pectin	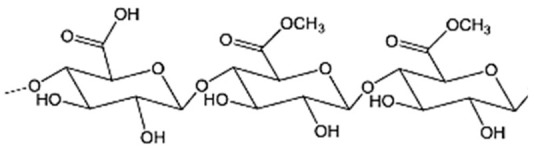	α-D-galacturonic acid is linked by α-(1-4)-glycosidic bonds and a variety of neutral sugars such as rhamnose, arabinose, and others. Pectins can be extracted in the form of acid, simple salt, esterified, methylated, or amidated, depending on the source and growth conditions.	Higher plant cell walls	[59]
Cellulose and derivatives	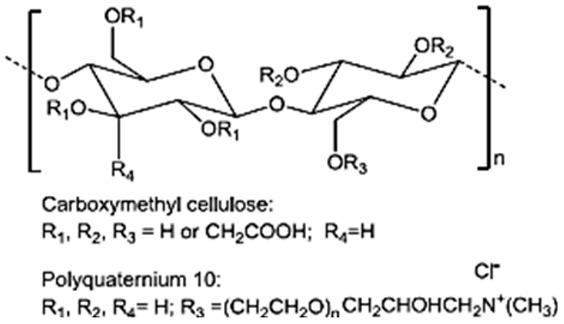	Anhydro-β-D-glucopyranose is linked by β-1,4-glycosidic bonds. Derivatives typically consist of semisynthetic ether or ester-substituted cellulose.	Higher plant cell walls and microorganisms	[59,62]
Starch	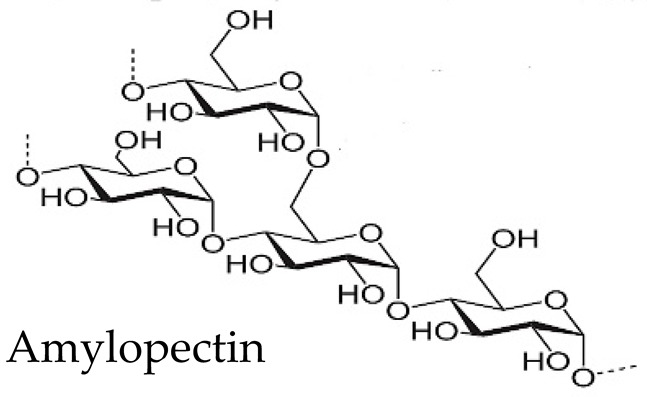	α-(1-4)-D-glucopyranose units are linked by α-1,4-glycosidic bonds. Amylose (lineal, 70%) and amylopectin (branched, 30%) chains	High variety of tubers, cereals, fruits, and stems	[63,64,65]
Konjac Glucomannan (KGM)	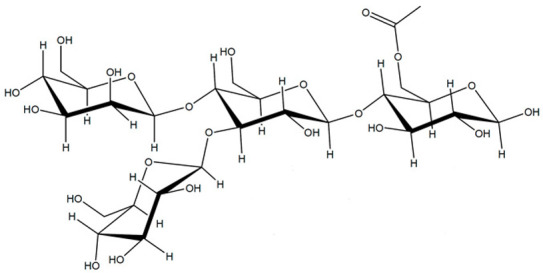	β-(1-4)-glucopiranose (G) and β-(1-4)-mannose units (M) are randomly linked by β-(1-4)-glycosidic bonds, with a ratio of 1:1.4–1.6 G/M ratios. Little ramifications in C3, and acetyl groups in C6 every 10–20 hexoses	Tubers of *Amorphophallus konjac*	[66,67,68]
Agar-Agar	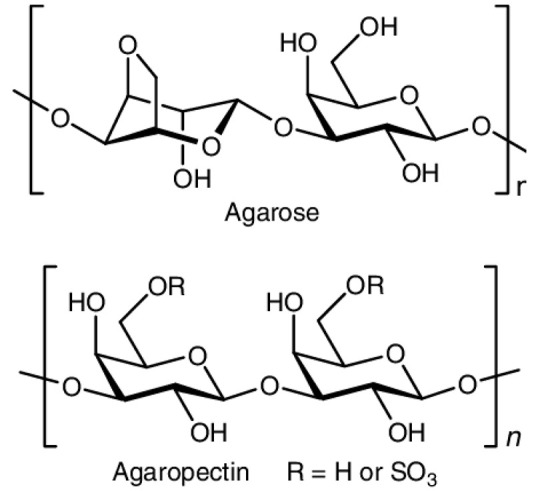	Agarose (70%, β-D-galactose and 3,6-anhydro-α-L-galactose units) and agaropectin (30%, β-D-galactose and L-galactose-6-sulfate groups). Units are linked by α-(1-3) and β-(1-4) glycosidic bonds.	Seaweeds from *Gracilaria* and *Gelidium* (red algae)	[59,69,70]
Xanthan gum	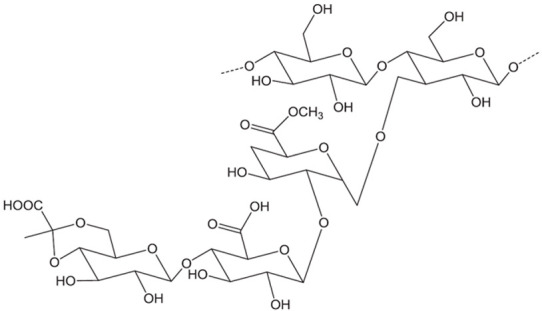	β-(1-4)-D-glucopiranose is linked by β-(1-4) glycosidic bonds, with trisaccharide chains of glucuronic acids on C3.	Fermentation processes of *Xanthomonas campestris*	[59,69]
Guar gum	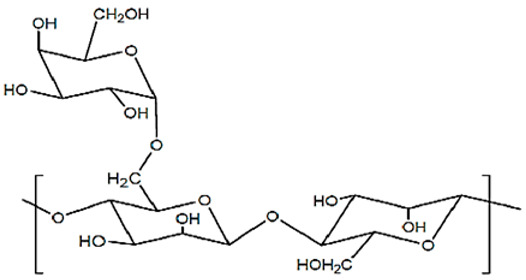	β-(1-4)-D-mannose (Man) is linked by β-(1-4) glycosidic bonds with α-(1-6)-D-galactopyranosyl (Gal) residues (1.37–2.0 Man/Gal ratio).	Seed endosperm of *Cyamopsis tetragonolobus*	[59,71]
Locust Bean gum	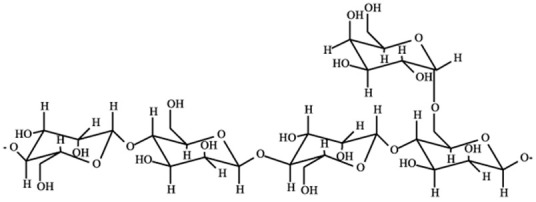	β-(1-4)-D-mannan is linked by β-(1-4) glycosidic bonds with branches of α-(1-6)-galactose	Seed endosperm of *Ceratonia silique*	[69,72]

**Table 3:** Overview of polysaccharide aerogel case studies over the past decade: production techniques and drug loading methods. For abbreviations’ explanations, please refer to Abbreviations.

Entry	Aerogel Composition and Gelation Technique	Drug	Preparation Method	Loading Method	Drug Release Conditions and Kinetic Models	Ref.
1	Potato starch (14.1% *w*/*v*). Gelation by retrogradation	Celecoxib	Sol–gel method in molds. Monolithic shapes.	Solvent exchange in drug-saturated ethanol solution	SGF (pH 1.2) for 7 h and SIF (pH 7.4) for 7 h Korsmeyer–Peppas model.	[111]
2	High-methoxyl pectin (1, 2, 4% *w*/*v*). Crosslinking by coagulation with EtOH.	Nifedipine	Sol–gel method in molds. Monolithic shapes	Solvent exchange in drug-saturated EtOH solution or supercritical impregnation	SGF (pH 1.2) for 1 h and PBS (pH 6.8) for 11 h. Korsmeyer–Peppas model. Higher drug release in PBS than SFG.	[112]
3	MCC. Gelation by non-solvent-induced phase separation.	Acetaminophen	Emulsion method and gelation in molds. Multiparticle and monolithic shapes.	Solvent exchange in drug-saturated EtOH solution	PBS (pH 7.4) at 37 °C. First-order kinetics release.	[113]
4	Alg, pectin, and mixtures (2% *w*/*v*). Ionic gelation (Ca^2+^, Zn^2+^, or Sr^2+^)	Diclofenac sodium	Dripping gelation. Multiparticle shapes.	Sol–gel dissolution	HCl media (pH 1.2), PBS (pH 6.8), and SGF. Korsmeyer–Peppas model.	[114]
5	Pectin. Ionic gelation (CaCl_2_ 0.5% *w*/*w*)	Vanillin	Jet cutting and dripping methods Multiparticle shapes.	SCF impregnation	Distilled water at 30, 40, and 50 °C. Weibull model.	[115]
6	Citrus pectin and MCC. Thermal and ionic gelation (CaCl_2_ 0.5 M)	Theophylline	Thermally induced gelation of MCC in molds, followed by pectin solution immersion. Monolithic shapes.	Solvent exchange in drug-saturated EtOH solution	SGF (pH 1.2) and SIF (pH 6.8) for 12 h. Korsmeyer–Peppas model. Formulations crosslinked with CaCl_2_ have longer sustained release than no crosslinked.	[116]
7	K-carrageenan, Alg, and reduced graphene oxide. Ionic gelation (CaCl_2_ 0.44% *w*/*w*)	Amoxicillin	Sol–gel method in molds. Monolithic shapes.	Sol–gel dissolution	Buffer (pHs 4.0, 5.5, 7.4 and 9.0). Korsmeyer–Peppas model. The cumulative drug release increases with the pH.	[117]
8	Alg 2% *w*/*w*. Ionic gelation (Ca^2+^ 4% *w*/*w*, and Ba^2+^ 4–12% *w*/*w*)	Ibuprofen	Dripping gelation. Multiparticle shapes.	Sol–gel dissolution	SGF (pH 1.2) and SIF (pH 7.2). Korsmeyer–Peppas release model	[118]
9	Pectin (2, 4 and 6% *w*/*v*). pH reduction and ionic gelation (CaCl_2_, Ca^2+^/COO^−^ ratios, from 0.05 to 0.2 according to the pectin concentration)	Theophylline	Sol–gel method in molds. Monolithic shapes	Solvent exchange in drug-saturated EtOH solution	SGF (pH 1.0) for 1 h, followed by SIF (pH 6.8) for 5 h. Peppas-Sahlin model: diffusional mechanism up to 60% released. Gallagher-Corrigan model: full release period	[119]
10	Alg 2% *w*/*v*, CS 1.5% *w*/*v*, and pectin 2% *w*/*v*. Gelation by non-solvent-induced phase separation (EtOH).	Esomeprazole	Sol–gel method in molds. Monolithic shapes	Solvent exchange in drug-saturated EtOH solution	SGF (pH 1.2) for 2 h and SIF (pH 6.8) for 2 h. Differences in release according to the loading procedure.	[120]
11	Kappa-carrageenan (2, 10% *w*/*v*). Ionic gelation (KCl 0.6 M, potassium thiocyanate 0.6 M, imidazolium cation).	Tetracycline	Sol–gel method in molds. Monolithic shapes	Impregnation in drug-saturated EtOH solution	Solution (pH 7.4) for 3 h. Korsmeyer–Peppas model. A total of 90% released in 60 min	[121]
12	Alg 2% *w*/*v* (low and high guluronic. Ionic gelation (Fe^3+^ 0.05 M).	Ibuprofen Ascorbic acid	Dripping gelation. Multiparticle shapes.	SCF impregnation	HCl media (pH 2.0) and PBS (pH 7.4). Korsmeyer–Peppas release model in acid medium	[122]
13	Silica, alg (1.5% *w*/*v*), pectin (6% *w*/*v*), or starch (15% *w*/*v*). Thermal or chemical gelation.	Ketoprofen or benzoic acid	Emulsion–gelation method. Multiparticle shapes	SCF impregnation	PBS (pH 6.8) and SGF (pH 1.2) for 24 h. Korsmeyer–Peppas and Gallagher-Corrigan models	[22]
14	Alg. Ionic gelation (Ca^2+^, Ba^2+^ 0.2 M).	Nicotinic acid and theophylline	Dripping gelation and additional gelation by immersion. Multilayer particles.	Sol–gel dissolution	SIF (pH 6.5) for 24 h. Korsmeyer–Peppas model. The number of layers increases the drug loading and the sustained release time	[123]
15	Alg. Ionic gelation (CaCl_2_ 0.3 M).	Ketoprofen and ketoprofen lysinate	Prilling (vibration, 300 Hz). Multiparticle shapes.	Sol–gel dissolution.	SGF (pH 1.2) for 2 h, and SIF (pH 6.8) for 4 h	[124]

**Table 4:** Materials and methods for preparing coated polysaccharide-based aerogels. For abbreviations’ explanations, please refer to Abbreviations.

Entry	Core Material	Coating Material	Coating Method	Advantages in Colonic Delivery	Reference
1	Alg aerogel. Ibuprofen loaded by SCF impregnation	Aqueous methacrylic acid-ethyl acrylate polymer solution	Wurster fluidized bed	pH response and controlled drug release	[131]
2	Cellulose aerogel. Vanillin loaded by SCF impregnation	Ethanol shellac solutions	Spouted bed	Controlled drug release	[132]
3	Alg–starch aerogels	Eudragit^®^ 30 D-55 (30% *w*/*v*)	Fluidized bed	pH response. Unconstrained water uptake at basic pH	[133]
4	Alg or cellulose aerogels	Perfluoro-acrylates	Cold plasma	Modulate the aerogel surface wettability	[134]
5	Alg beads	Alg solutions (0.75% *w*/*v*)	Multi-step sol–gel process	Controlled release of hydrophilic drug	[135]
6	Pectin solution	Alg solution (1.5–1.75% *w*/*v*)	Coaxial prilling	Sustained drug release	[136]

## Abbreviations

Alg: alginate; AU: commercial apple pectin; CaCl_2_: calcium chloride; CA: hybrid calcium alginate; CaCO_3_: calcium carbonate; CFU: colony-forming unit; CMC: carboxymethyl cellulose; CO_2_: carbon dioxide; CS: chitosan; DA: degree of amidation; DD: deacetylation degree; DE: degree of esterification; DNA: deoxyribonucleic acid; ENA: Aquarius™ Control ENA coating polymer; EtOH: ethanol; FDA: Food and Drug Administration; GIT: gastrointestinal tract; GRAS: Generally Recognized As Safe; HCl: hydrochloric acid; H-HPC: high substitute hydroxypropyl cellulose; HPMCP: hydroxypropyl methylcellulose phthalate; IBD: inflammatory bowel diseases; KGM: konjac glucomannan; LMC: *Lemna minor callus* pectin; MCC: microcrystalline cellulose; NIPS: non-solvent-induced phase separation; NME: new molecular entity; MW: molecular weight; NP: nanoparticle; ODT: oral disintegrating formulation; PBS: phosphate-buffered saline; ROS: reactive oxygen species; scCO_2_: supercritical carbon dioxide; SCF: supercritical fluid; SCFAs: short chain fatty acids; SCoF: Simulated Colonic Fluids; SGF: simulated gastric fluid; SIF: simulated intestinal fluid; SVC: *Silene vulgaris callus* pectin; W/O: water-in-oil.
